# Fgfr1 Inactivation in the Mouse Telencephalon Results in Impaired Maturation of Interneurons Expressing Parvalbumin

**DOI:** 10.1371/journal.pone.0103696

**Published:** 2014-08-12

**Authors:** Karen Müller Smith, Maria Elisabetta Maragnoli, Pooja M. Phull, Kathy May Tran, Lisha Choubey, Flora M. Vaccarino

**Affiliations:** 1 Child Study Center, Yale University, New Haven, Connecticut, United States of America; 2 Department of Biology, University of Louisiana at Lafayette, Lafayette, Louisiana, United States of America; 3 Department of Neurobiology, Yale University, New Haven, Connecticut, United States of America; 4 Kavli Institute for Neuroscience, Yale University, New Haven, Connecticut, United States of America; Washington University, School of Medicine, United States of America

## Abstract

Fibroblast growth factors (Fgfs) and their receptors (Fgfr) are expressed in the developing and adult CNS. Previous studies demonstrated a decrease in cortical interneurons and locomotor hyperactivity in mice with a conditional Fgfr1 deletion generated in radial glial cells during midneurogenesis (Fgfr1^f/f^;hGfapCre+). Here, we report earlier and more extensive inactivation of Fgfr1 in neuroepithelial cells of the CNS (Fgfr1^f/f^;NesCre+). Similar to findings in Fgfr1^f/f^;hGfapCre+ mice, parvalbumin positive (PV+) cortical interneurons are also decreased in the neocortex of Fgfr1f/f;NesCre+ mice when compared to control littermates (Fgfr1f/f). Fgfr1f/f;NesCre+ embryos do not differ from controls in the initial specification of GABAergic cells in the ganglionic eminence (GE) as assessed by in situ hybridization for Dlx2, Mash1 and Nkx2. Equal numbers of GABAergic neuron precursors genetically labeled with green fluorescent protein (GFP) were observed at P0 in Fgfr1^f/f^;hGfapCre+;Gad1-GFP mutant mice. However, fewer GFP+ and GFP+/PV+ interneurons were observed in these mutants at adulthood, indicating that a decrease in cortical interneuron markers is occurring postnatally. Fgfr1 is expressed in cortical astrocytes in the postnatal brain. To test whether the astrocytes of mice lacking Fgfr1 are less capable of supporting interneurons, we co-cultured wild type Gad1-GFP+ interneuron precursors isolated from the medial GE (MGE) with astrocytes from Fgfr1f/f control or Fgfr1^f/f^;hGfapCre+ mice. Interneurons grown on Fgfr1 deficient astrocytes had small soma size and fewer neurites per cell, but no differences in cell survival. Decreased soma size of Gad67 immunopositive interneurons was also observed in the cortex of adult Fgfr1^f/f^;NesCre+ mice. Our data indicate that astrocytes from Fgfr1 mutants are impaired in supporting the maturation of cortical GABAergic neurons in the postnatal period. This model may elucidate potential mechanisms of impaired PV interneuron maturation relevant to neuropsychiatric disorders that develop in childhood and adolescence.

## Introduction

The mammalian cerebral cortex is a six-layered laminar structure formed by neurogenic stem cells situated in the ventricular and subventricular zones of the dorsal telencephalon that proliferate and give rise to excitatory neurons and astrocytes. GABAergic inhibitory interneurons and oligodendrocytes that arise in the ventricular zone of the ganglionic eminence (GE) also contribute to cortical circuitry. Several fibroblast growth factor (Fgf) ligands and three Fgf receptors (Fgfr1, Fgfr2, and Fgfr3) are expressed in the developing telencephalon. Fgfs are powerful mitogens for neuroepithelial stem cells, delay neural stem cell differentiation, in part via Notch signaling [1], and regulate area-specific cortical growth and dorso-ventral patterning of the forebrain [2]–[7]. Previously, we generated CNS specific conditional mutants of the Fgfr1 gene by Cre mediated recombination of floxed Fgfr1 alleles driven by the human glial fibrillary acidic protein (hGFAP) promoter (hGfapCre+;*Fgfr1* ^f/f^ mice). The hGFAP-Cre transgene targets Cre protein primarily to radial glial progenitors of the dorsal telencephalon beginning at mid neurogenesis, after major CNS patterning events have occurred, and after the initial formation of the cortical plate. The hGfapCre+;*Fgfr1* ^f/f^ mice have a primarily midline phenotype, including a decrease in the size of the hippocampus, a lack of glial cell soma translocation in the dorsal midline, resulting in agenesis of the indusium griseum and corpus callosum. Surprisingly, these mutants also exhibited a decrease of cortical inhibitory interneurons as assessed by unbiased stereology of immunolabeled brain sections [5], [8], [9].

Cortical interneurons are a diverse group of cells expressing the neurotransmitter gamma-amino butyric acid, or GABA, and are important regulators of neuronal excitability and synaptic plasticity, facilitating the organization of excitatory neurons into functional units, and the coordinated firing of groups of excitatory neurons [10]–[12]. Cortical GABA interneurons have been classified into various subsets based on their expression of parvalbumin (PV), calretinin (CR), Neuropeptide Y (NPY), vasoactive intestinal peptide (VIP), reelin and somatostatin (Sst) [13]–[15]. Electrophysiological subtypes include fast spiking interneurons, burst spiking non-pyramidal cells, regular spiking non-pyramidal cells, late spiking cells, and irregular spiking cells [16]. These classification systems can be integrated to classify interneurons into specific subtypes. For example, PV+ chandelier interneurons are fast-spiking cells that make axonal contact onto the cell body and proximal axonal segment of excitatory neurons [12], [16].

hGfapCre+;*Fgfr1* ^f/f^ and *Fgfr2* ^f/f^ mice have significant decreases in cortical and hippocampal interneurons expressing PV, but do not lack CR interneuron subtypes [9], [17]. Furthermore, the reduction in cortical PV interneurons was correlated to levels of hyperactivity in hGfapCre+;*Fgfr1* ^f/f^ mice [9]. In order to understand the mechanisms and timing by which Fgfr1 affects the GABAergic neuron system during telencephalic development, we generated conditional Fgfr1 mutants with the Cre recombinase driven by the Nestin promoter (*Fgfr1^f/f^;Nestin-Cre+* mice), which has been shown to drive Cre activity to the entire CNS starting from E10.5 [18]. Here, we show that the *Fgfr1^f/f^;Nestin-Cre+* mice share many features of *Fgfr1^f/f^;hGFAP-Cre+* mice, including a severe deficit in PV+ cortical interneurons. We find no evidence for a disruption in the early development of cortical interneurons in these mice, suggesting a role for Fgfr1 in the postnatal maturation, and/or survival of cortical interneurons. Immunoassays and RT-PCR for KV3.1b, Lhx6, and PV indicated that parvalbumin interneuron numbers and transcript levels were unchanged, but that rather, PV protein levels were decreased. By mating *Gad67-GFP* knock-in to Fgfr1 mutant lines, we were able to trace the prenatal and postnatal development of GABA-containing interneurons, which further support a postnatal mechanism for the decrease in PV and GFP expression and altered interneuron maturation in Fgfr1 mutant animals. Furthermore, co-culture experiments indicate that Fgfr1 mutant astrocytes provide deficient trophic support for interneurons.

## Materials and Methods

### Animals

The genetically modified mouse lines, Tg(Nes-Cre), Tg(GFAP-Cre)25Mes (referred to here as hGFAP-Cre), Fgfr1^flox/flox^, and mating strategies have been previously described [9]. Gad67-GFP mice (kindly provided by Yuchio Yanagawa), were mated to the Fgfr1^flox^ mice, to generate Gad67-GFP+; Fgfr1^flox/flox^ mice. Gad67-GFP+; Fgfr1^flox/flox^ mice were bred to *Fgfr1^f/f^;Nestin*-*Cre+* and *Fgfr1^f/f^;hGFAP-Cre+* to generate Fgfr1 mutants with the Gad67-GFP allele. The GENSAT project (GENSAT.org) generated line Tg(Fgfr1-EGFP)GP338Gsat was obtained from the Mutant Mouse Resource Center (MMRRC.org) at UC Davis. This study was carried out in strict accordance with the recommendations in the Guide for the Care and Use of Laboratory Animals of the National Institutes of Health. The protocol was approved by the Yale University Institutional Animal Care and Use Committee (protocol number 2012-07621); some animals were euthanized under UL Lafayette IACUC committee APS number 2013–8717–053. All tissue collection was performed under ketamine/xylazine cocktail or isoflurane mixture anesthesia, and all efforts were made to minimize suffering.

### In situ hybridization and immunohistochemistry

Digoxigenin-labeled RNA probes were synthesized from cDNAs by *in vitro* transcription (Digoxigenin RNA labeling kit, SP6/T7, Roche, Germany) using previously described techniques. Primary antibodies (**Table 1**) for immunohistochemistry were detected with Alexa conjugated secondary antibodies (Molecular Probes, Jackson labs) or AMCA conjugated secondary antibodies (Vector) for fluorescent detection. For the analyses of cell proliferation, pregnant dams were injected with 2-bromodeoxyuridine (BrdU) was injected (100 µg/gr, i.p.) at E13.5, and sacrificed 24 hours later at E14.5 by cervical dislocation of the mother, under anesthesia, or at P0 by decapitation after anesthesia by hypothermia. Embryos were harvested immediately and fixed in 4%PFA. BrdU was detected as previously described.

**Table 1:** Antibodies used for Immunohistochemistry and Western Blotting.

Antigen	Raised in	Dilution	Source
APC	Mouse	1∶200	Calbiochem
Activated Caspase	Rabbit	1∶500	Cell Signaling
B-actin	mouse	1∶4000	Sigma
BDNF	Rabbit	1∶500	Santa Cruz
BrdU	Rat	1∶500	Accurate Chemical
Calretinin	Rabbit	1∶5000	Swant
Dlx2	Guinea Pig	1∶9000	Dr. Yoshikawa [77]
Fgfr1 (YU302)	Rabbit	1∶1000	Dr.Schlessinger [78]
Gad67	Mouse	1∶2000	Millipore/Chemicon
GLAST	Guinea Pig	1∶2000	Millipore/Chemicon
GFP	Rabbit	1∶1000	Invitrogen
GFP	Chicken	1∶1000	Abcam
GFAP	Rabbit	1∶1000	DAKO
GFAP	Mouse	1∶500	Sigma
HGF	Goat	1∶500	R&D
MASH1	Mouse	1∶100	Götz Lab [79]
Ki67	Mouse	1∶300	NovoCastra
KV3.1b (KCNC1)	rabbit	1∶500	Alomone
NG2	Mouse Monoclonal	1∶250	Chemicon
NeuN	Mouse Monoclonal	1∶500	Chemicon
Parvalbumin	Mouse Monoclonal	1∶1000–1∶2500	Sigma
PSD95	Rabbit	1∶250	Invitrogen
Olig2	Rabbit	1∶20,000	Dr. Stiles [80]
S100 beta	Rabbit	1∶400	Sigma
Somatostatin	Rat	1∶200	Millipore/Chemicon
Tbr1	Rabbit	1∶1000	Dr. Englund and Dr. Hevner [81]

### Immunoblotting

Mice (7 week old) were euthanized by cervical dislocation under anesthesia, the brains were dissected out, and the cortex and hippocampus were isolated by microdissection. Tissue was lysed in a glass homogenizer in lysis buffer containing: 50 mM Tris-HCL pH = 7.4, 150 mM NaCl, 50 mM NaF, 1% SDS, 1% NP-40, 0.5% w/v Sodium Deoxycholate, 1 mM Sodium Orthovanadate, 0.2 mM Sodium Pyrophosphate, and protease inhibitor cocktail with EDTA (Roche). Homogenates were centrifuged at 13,000 RPM for 10 minutes at 4°C and supernatants were stored at −80°C until used for 10–15% sodium dodecyl sulfate polyacrylamide gel electrophoresis (SDS-PAGE). Proteins were transferred to a nitrocellulose membrane, and membranes were blocked with blocking buffer (5% nonfat milk in Tris buffered saline with 0.1% Tween-20, or TBST). Membranes were incubated overnight with primary antibodies in blocking buffer, washed with TBST, and incubated with horseradish peroxidase conjugated secondary antibodies (1∶3,000 to 1∶10,000 sheep anti mouse (GE, Healthcare), 1∶5:000 Donkey anti Rabbit or 1∶5000 goat anti guinea pig (Jackson Labs). Membranes were washed with TBST and detected with Super Signal West Femto Chemiluminescent Substrate from Pierce on a G-Box instrument from Syngene. Blots were stripped with one-minute stripping buffer (GM biosciences).

### RNA isolation and RT-PCR

One month old mice (3 Fgfr1^f/f^ control and 4 Fgfr1^f/f^;Nestin-Cre+), or four month old mice (3 Fgfr1^f/f^ control and 3 Fgfr1^f/f^;Nestin-Cre+) were euthanized as described above and whole cortical tissue was homogenized in Trizol reagent (Life Technologies) to isolate RNA. RNA was quantified on a nanodrop spectrophotometer, and equal amounts of template were converted to cDNA with the High Capacity cDNA Reverse Transcriptase Kit (Life Technologies). QRT-PCR was performed with a StepOne Plus instrument using the TaqMan Universal Master Mix II, with uracil-N glycosylase (UNG). For KV3.1b the assay identification was KCNC1 Mm00656608_m1 (Life Technologies) and the reference gene assay was mouse B-actin Mm pt 39a22214843 (IDT technologies). The following commercially available assays were performed: KCNC1 Mm00656608_m1 for KV3.1b (Life Technologies), Pvalb Mm00443100_m1 for PV (Life Technologies), Lhx6 Mm01333348_m1 (Life Technologies), and the reference gene assay was mouse B-actin Mm pt 39a22214843 (IDT technologies).

### Cell counting

Unbiased estimates for total cell number were obtained using a computer coupled to a Zeiss Axioskope 2 Mot Plus equipped with a motorized stage, running the StereoInvestigator software (Microbrightfield). Nuclear profiles were counted in 3-dimensional counting boxes, using a randomly placed sampling grid of 750×750 µm (Sst), 1000×1000 µm or 1250×1250 µm on the adult cerebral cortex. Counting boxes, 85×70×15 µm (for Sst), or 100×100×5 µm, were placed 0.5 µm below the surface at each of the grid intersection points. Cells were only counted if they overlapped a DAPI-stained cell nucleus. For counts in the adult cortex, 50 µm coronal sections were counted at a frequency of one every 20^th^ section. Cerebral cortical contours were drawn at the caudal border of layer 6 and included the cingulate cortex and neocortex, but excluded the archicortex with a boundary located at the entorhinal/hippocampal border. For counts of the cerebral cortex at P0, 20 µm sections were sampled every 20^th^ section. For BrdU and GFP counts, a sampling grid of 800×800 µm was used and a counting box of 75×75×5 µm was placed 0.5 µm below the surface. For Activated Caspase 7 at P0, a sampling grid of 500×500 µm was used and a counting box of 200×200×5 µm was placed 0.5 µm below the surface. For Activated Caspase 7 and GFP counts of the cortex at P7, 50 µm coronal sections were sampled every 10^th^ section with a sampling grid of 700×700 µm. Counting Boxes were 300×200×15 µm. For counts of the MGE, 20 µm coronal sections were counted every 10^th^ section. The MGE was demarcated between the LGE and CGE. A sampling grid of 200×200 µm was used and a counting box of 50×50×5 µm was placed 0.5 µm below the surface. Fluorescent images were obtained on a Zeiss axiovert microscope equipped with an apotome system and an Orca-ER camera, or a Zeiss Imager M2 microscope equipped with an apotome system and an Axiocam MRm camera.

### Astrocyte/Interneuron co-culture experiments

Astrocytes were harvested from P2-P4 pups of hGfapCre+;*Fgfr1* ^f/f^ or control littermate *Fgfr1* ^f/f^ pups (at least three samples per group). Pups were anesthetized by hypothermia and tail samples for DNA were collected on anesthetized pups prior to decapitation. Decapitated heads were rinsed 1× with ethanol, followed by HBSS (No Calcium, no Magnesium, Life Technologies). Brains were dissected out in cold HBSS; care was taken to remove meninges prior to isolating the cortical plate by microdissection under a dissecting microscope. Brains were kept cold by placing the dissection dish with cold HBSS on an icepack. Cortical tissue was first chopped with a sterile razor blade to about 0.5 mm cubes prior to trypsin digestion. The pieces of cortex in HBSS were transferred to a 15 ml conical tube with a sterile transfer pipette. Once all brains were collected and chopped, the samples were spun at 600 RCF, 4°C to collect pieces of cortex. Under a laminar flow hood, 2 ml of 0.25% trypsin/EDTA (Life Technologies) was added and tubes were tapped to a tabletop shaker/incubator at 37°C and 300 RPM for 12 minutes. The trypsin was inactivated with 2 ml of DMEM/F12/10% FBS (Life Technologies) and DNAseI was added (0.1 mg/ml Roche) to digest DNA in the solutions and facilitate collecting cells by centrifugation. Samples were centrifuged at 600× RCF, 4°C, the supernatant was aspirated, and then resuspended in HBSS 0.1 mg/ml DNAseI. The tissue was triturated 15 times with trituration pipettes prepared by fire polishing the opening of a plugged borosilicate glass pipette. The suspension was allowed to settle for 10 minutes and then the cell suspension in the supernatant was transferred to a new conical tube. Trituration was repeated on any remaining tissue pieces that were left behind and cell suspensions were combined prior to collection of the cells by centrifugation at 600 RCF. Collected cells were resuspended in DMEM/F12/10%FBS with penicillin/streptomycin (Life Technologies) and plated in an uncoated T75 flask. All cells from each brain were grown in one flask in a humidified incubator at 37°C and 5%CO2. Media was changed the next day, and then every 3–4 days. Flasks were split by trypsinization when they achieved confluence. For co-culture experiments, the astrocytes were trypsinized from a nearly confluent flask, and plated at a cell density of 5×10^4^ cells/well of a 4 well cell culture slide and grown for 3 days prior to co-culture with interneuron precursors. Interneurons were isolated from the MGE of E13.5 embryos with the GAD67-GFP allele from timed pregnancies (midday that the plug was found was designated E 0.5). Pups positive for GFP were screened by fluorescence. The brains were dissected as above in HBSS under a dissection microscope. The MGE was removed using fine angled forceps, chopped with a sterile blade as above and directly triturated in HBSS with DNAseI as described above. Triturated cells were resuspended at a density of 20×10^4^ cells in Neurobasal media with 1 ml B27, 5%Glucose, Pen/strep and L-glutamine (Neurobasal/B27). The DMEM/F12/10% FBS media was removed from the astrocytes and replaced with 0.7 ml of Neurobasal/B27 media and 0.5 ml of the MGE cell preparation (10×10^4^ cells total). Cells were co cultured for 14 days, or for 21 days in the presence of 30 µM KCl. Cells were fixed with fresh 4%PFA/1×PBS at the time of harvest and stained as described above for tissue sections.

For cell tracing experiments, 12 cells per sample from at least three wells were traced using the Neurolucida program (MBF) for a total of 60 neurons grown on Control Astrocytes (five independent animals) and 38 neurons grown on Fgfr1 mutant astrocytes (three independent animals), from three separate experiments. GFP positive interneurons were traced at 100×, with the axon identified by morphological characteristics including a non-tapering morphology. For cell counts of co-cultures, the StereoInvestigator program (MBF) was adapted to trace the wells of the culture chamber and then place 100×100 µm boxes in a 1500×1500 µm grid in each well using the fractionator function. These data were used to estimate the number of cells per well.

### Analysis of PSD95 and Gad67 colocalization

Two pictures of layers IV–VI of the dorsal neocortex from Z stacks of 50 um coronal sections of control and Fgfr1 mutant animals, three animals per group, were obtained on a Zeiss axiovert microscope equipped with an apotome system and an Orca-ER camera. Areas were matched by using cytoarchitectual landmarks. The first picture was obtained above the white matter in the motor cortex above the caudate putamen, lateral ventricle, septum and BST, and anterior to the fimbria. The second picture was taken of the somatosensory cortex, at the level where the dorsal hippocampus and dentate gyrus appears, posterior to the fimbria. Images were imported to Image J, and the Gad67 channel was used to identify and guide the tracing of Gad67 immunopositive cells. The average signal intensity within the traced regions of the Psd95 channel was determined, as was the average area within the traced regions. Sixty cells from control samples and 78 cells from Fgfr1 mutant samples were analyzed, and values were averaged for each picture.

### Statistical Analysis

Data were first entered into excel then imported to statistical software and analyzed by student t-tests or ANOVA using SAS or SPSS. Sequential Bonferroni correction was applied in cases of multiple sampling. For the analysis of Neurolucida results, each neuron was considered an independent sample of two measures (cell body perimeter and number of neurites); the measures were found to be significant and the post hoc analysis found these measures to be significantly correlated to each other. Therefore, ANCOVA analysis was performed to determine whether group effects were attributable to the covariation of these two factors, or whether each of these factors were independently significant. The number of samples is specified in each case in the figure legends.

## Results

### Disruption of *Fgfr1* by the *Nestin-Cre* transgene results in a loss of parvalbumin positive interneurons

**Figure 1:**
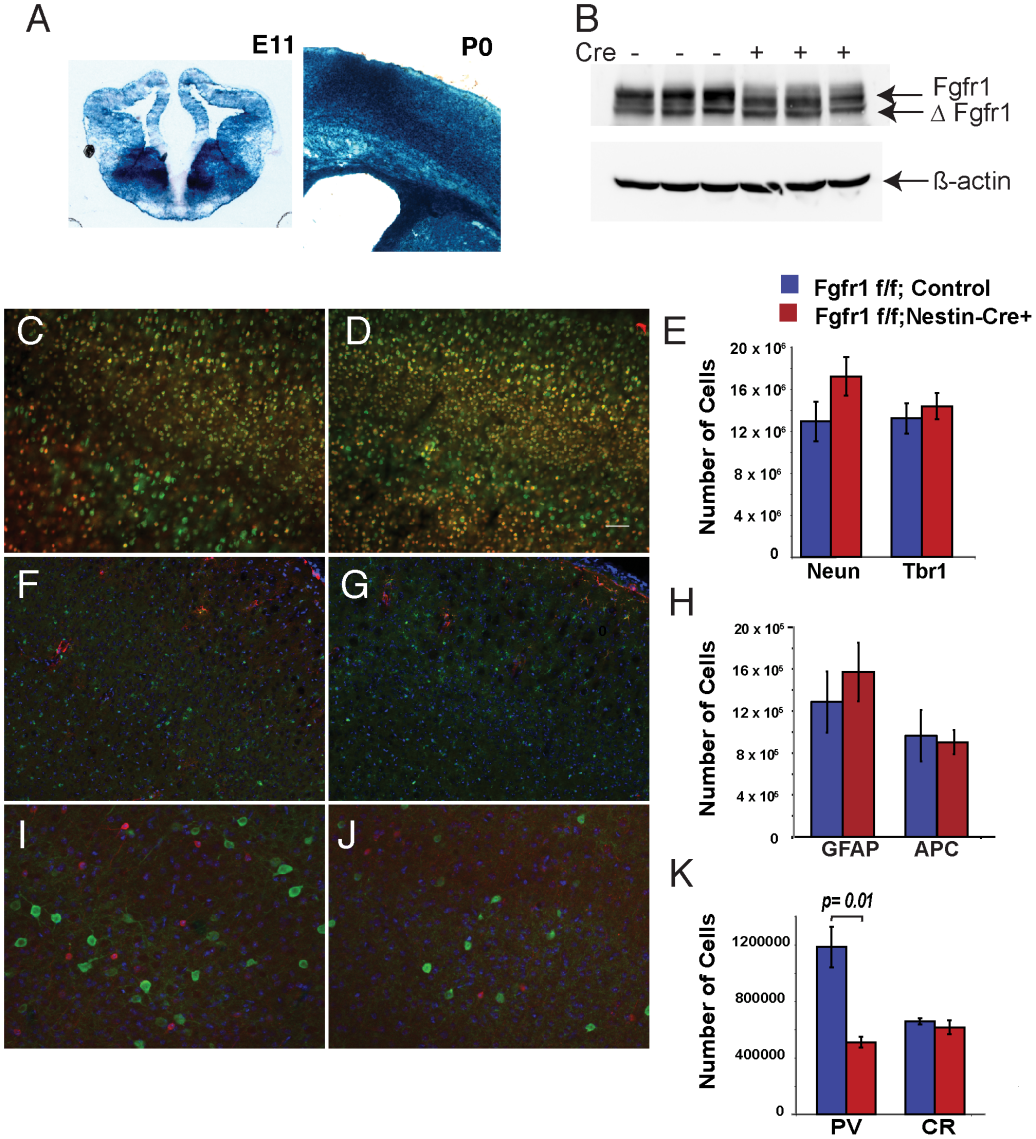
Deletion of Fgfr1 in developing telencephalon results in decreased numbers of PV+ interneurons. Nestin-Cre mediated recombination of the ROSA R26R locus in the developing CNS demonstrates Cre activity in the ganglionic eminences and in the dorsal telencephalon at E11 and P0 (**A**). Immunoblotting for Fgfr1 identifies a smaller protein product and reduced full-length protein in Fgfr1 mutant animals at 7 weeks of age (average signal relative to beta actin was 4.51 in Control vs 1.79 vs Fgfr1^f/f^;Nestin-Cre+, p = 0.03, **B**). No differences in the number of Neun (green), Tbr1 (red) (**C**,**D**), GFAP (red) or APC (green) (**F**,**G**) positive cells were observed in the cerebral cortex of Two month old control and mutant animals (**C–H**). The number of cortical PV+ interneurons (green) was reduced in *Fgfr1^f/f^;Nestin-Cre+* mice compared to *Fgfr1^f/f^* control mice (**I–K**) (n = 3 per group), but calretinin positive interneurons (red) were unchanged. Scale bar is 50 µm in **C,D,F,G**, and 25 µm **I**,**J**.

We previously reported a decrease in PV positive interneurons in conditional mutants of Fgfr1 driven by the *hGFAP-Cre* transgene [9]. We examined whether this phenotype is more severe if more extensive and earlier disruption of *Fgfr1* is achieved with the *Nestin-Cre* transgene in Fgfr1^f/f^;Nestin-Cre+ mice [8]. The Nestin Cre transgene targets the entire CNS beginning at E10.5, at least 3 days earlier than the hGFAP-Cre transgene. The recombination reporter mice *ROSA R26R* mated to the mice with the *Nestin Cre* transgene demonstrates extensive *lac-Z* reporter recombination in both the dorsal and basal telencephalon at E11.5 (**Figure 1A**). Immunoblot analysis confirmed that the *Fgfr1* gene was successfully recombined in the dorsal telencephalon of Fgfr1^f/f^;Nestin-Cre+ mice with a smaller protein product and reduced signal for full-length protein in Fgfr1 mutant animals (**Figure 1B**).

We examined several neuronal and glial cell types by immunohistochemistry and unbiased stereology of 2 month-old Fgfr1^f/f^;Nestin-Cre+ animals and their control *Fgfr1^f/f^* littermates. The transcription factor TBR1 is expressed by excitatory neurons in layers 6 and 2–3. As was found with the *Fgfr1^f/f^;hGFAP-Cre+* mutant mice, there were no significant differences in the pan-neuronal marker Neun or in TBR1 positive cells in *Fgfr1^f/f^;Nestin-Cre+* mice as compared to their littermate controls (**Figure 1C–E**). Furthermore, no differences in astrocytes labeled by GFAP, or in oligodendrocytes labeled with the APC antibody were detected between groups (**Figure 1F–H**).

A significant decrease in PV+ neurons was observed in *Fgfr1^f/f^;Nestin-Cre+* mice as compared to their littermate controls. However, there was no significant decrease in CR+ interneurons in these mutant mice (**Figure 1I–K**). The 60% decrease in PV+ interneurons was more extensive than that found for *Fgfr1^f/f^;hGFAP-Cre+* mutants, which was about 30% [9]. A decrease in PV protein by immunoblotting was also detected in Fgfr1 mutants (**Figure S1A,B**). KV3.1b is a potassium channel subunit reported to be specifically expressed by PV neurons [19], [20]. However, no differences in KV3.1b protein were detected between control and Fgfr1 mutants (**Figure S1C**). We also performed RT-PCR for KV3.1b and found no significant differences between *Fgfr1^f/f^* control and *Fgfr1^f/f^;Nestin-Cre+* at 1 month or 4 months of age (**Figure S2J**,**K**). However, immunostaining revealed that not all KV3.1b positive cells in the cortex were also PV positive (**Figure S2A–C**,**E–G**). Next, we performed immunostaining (**Figure S2D**,**H**) and stereological analyses of somatostatin-positve (Sst+) interneurons. We found 416,403±103,933 cells (n = 3) in *Fgfr1^f/f^* controls and 275,897±42,848 (n = 3) in *Fgfr1^f/f^;Nestin-Cre+* mice. While reduced in number, Sst+ interneurons were not statistically significant between mutants and controls (p = 0.28, **Figure S2I**).

To examine whether the number of PV+ interneurons was indeed reduced, rather than not expressing sufficient amounts of PV, we performed quantitative RT-PCR assays for the transcription factor Lhx6 (which is necessary for the PV interneuron cell fate, and which marks PV neurons), and PV itself (**Figure S2L,M**). Expression of Lhx6 and PV was not detectibly altered at the mRNA level, suggesting that the interneurons are still present, but with altered regulation of PV protein levels. Importantly, RT-PCR did detect decreased levels of Fgfr1 mRNA in *Fgfr1^f/f^;Nestin-Cre+* mice (**Figure S2N**).

### Patterning of the Medial Ganglionic Eminence (MGE) is preserved in Fgfr1 mutant animals

**Figure 2:**
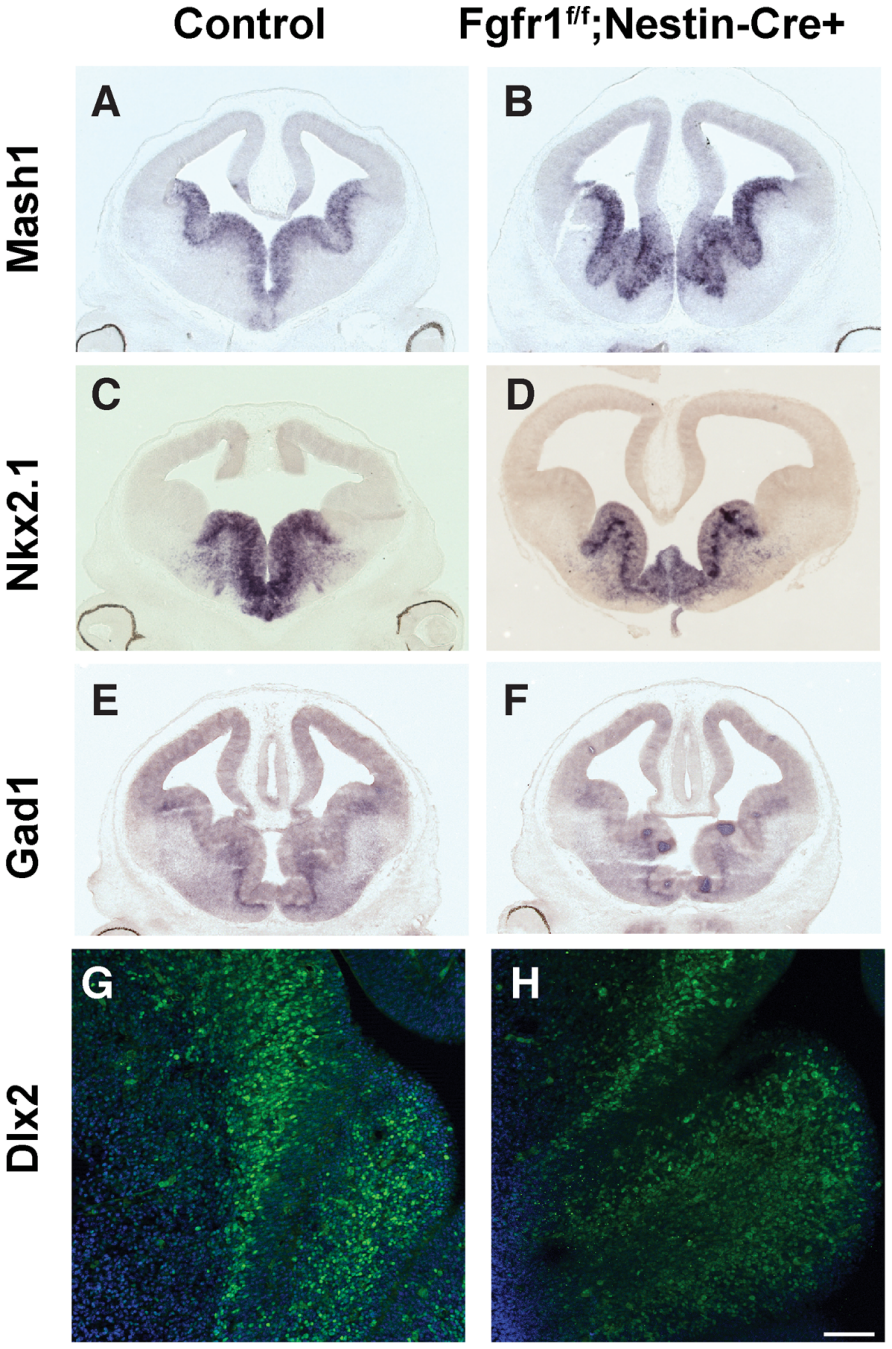
Fgfr1 mutation does not alter the initial patterning of the ganglionic eminence and specification of the interneuron lineage. *In situ* hybridization for *Mash1* (**A**,**B**) and *Nkx2.1* (**C**,**D**), in controls (**A,C**) and *Fgfr1^f/f^;Nestin-Cre+* mutant mice (**B,D**), revealing that gene expression for these cell fate determinants for the PV neuron lineage was not disrupted by Fgfr1 mutation at E12.5. *In situ* hybridization for *Gad1* demonstrated that GABA synthetic enzymes are appropriately expressed in Fgfr1 (**F**) mutants compared to controls (**E**) at E12.5. Immunofluorescence staining for the transcription factor Dlx2, also revealed equivalent staining in the MGE of control (**G**) and Fgfr1 mutants (**H**). Scale bar 20 µm in G and H.

PV positive interneurons of the cortex are derived from the ventral telencephalon, and specifically from the MGE. As the patterning of the ventral telencephalon is influenced by secreted morphogens such as Shh and Fgf [21], we examined whether deletion of the Fgfr1 gene results in abnormal patterning of the MGE. In situ hybridization of the Mash1 and NKX2.1 was performed in E13.5 embryos. This analysis revealed that *Fgfr1^f/f^;Nestin-Cre+* mice had comparable distribution of both Mash1 and Nkx2.1 mRNA, and therefore normal patterning of the MGE (**Figure 2A–D**). Furthermore, in situ hybridization for the Gad-1 transcript was similar in both groups, showing that early expression of the GABAergic cell fate is preserved in Fgfr1 mutant animals (**Figure 2E,F**). Finally, Dlx2 immunofluorescence at E14.5 showed no differences in expression patterns in control *Fgfr1^f/f^ or Fgfr1^f/f^;Nestin-Cre+* mice (**Figure 2G,H**).

### Proliferation and migration of interneuron precursors is preserved in Fgfr1 mutant animals

**Figure 3:**
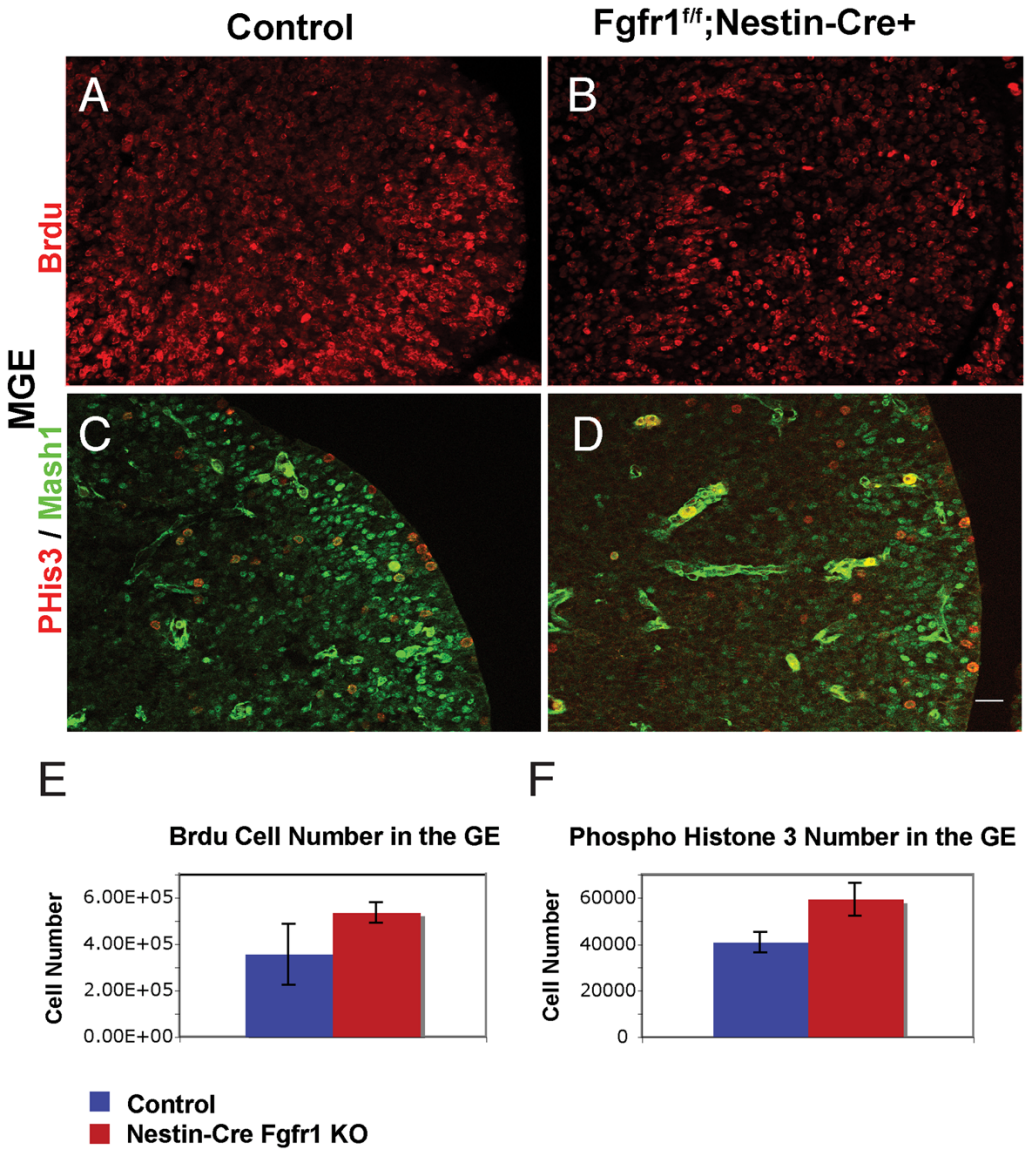
Cell proliferation in the MGE is not altered in Fgfr1 mutants at E13.5. BrdU labeling in the MGE of *Fgfr1^f/f^* Control (**A**) and *Fgfr1^f/f^;Nestin-Cre* (**B**), and Phospho-histone 3 (PHis3) and Mash1 immunolabeling in the MGE of *Fgfr1^f/f^* Control (**C**) and *Fgfr1^f/f^;Nestin-Cre* (**D**) embryos. BrdU was administered at E13.5, and embryos were analyzed at E14.5. No significant differences in the number of BrdU labeled (**E**) or PHis3 labeled cells (**F**) were observed by stereology (n = 3 for each group). Scale bar is 25 µm.

Fgf ligands are known to have mitogenic effects upon neural stem cells via Fgfr signaling. Therefore, we examined whether cell proliferation in the GE was altered in Fgfr1 mutant animals. We administered 2-bromodeoxiuridine (BrdU) to pregnant dams at E13.5, and examined the number of BrdU labeled cells in the GE of Fgfr1 mutant animals. There was no significant difference in the BrdU labeling index between *Fgfr1^f/f^;Nestin-Cre+* and their littermate controls (p = 0.26, **Figure 3A,B, E**). We did observe a non-significant trend of increased numbers of phosphohistone 3+ cells, a marker for actively mitotic cells, in the GE of *Fgfr1^f/f^;Nestin-Cre+* mice (p = 0.09, **Figure 3C,D,F**). No significant differences in cell proliferation were observed, even though Nestin-Cre targeting of the GEs is evident by ROSA R26R staining by E11.5 in mutant animals (**Figure 1A**).

**Figure 4:**
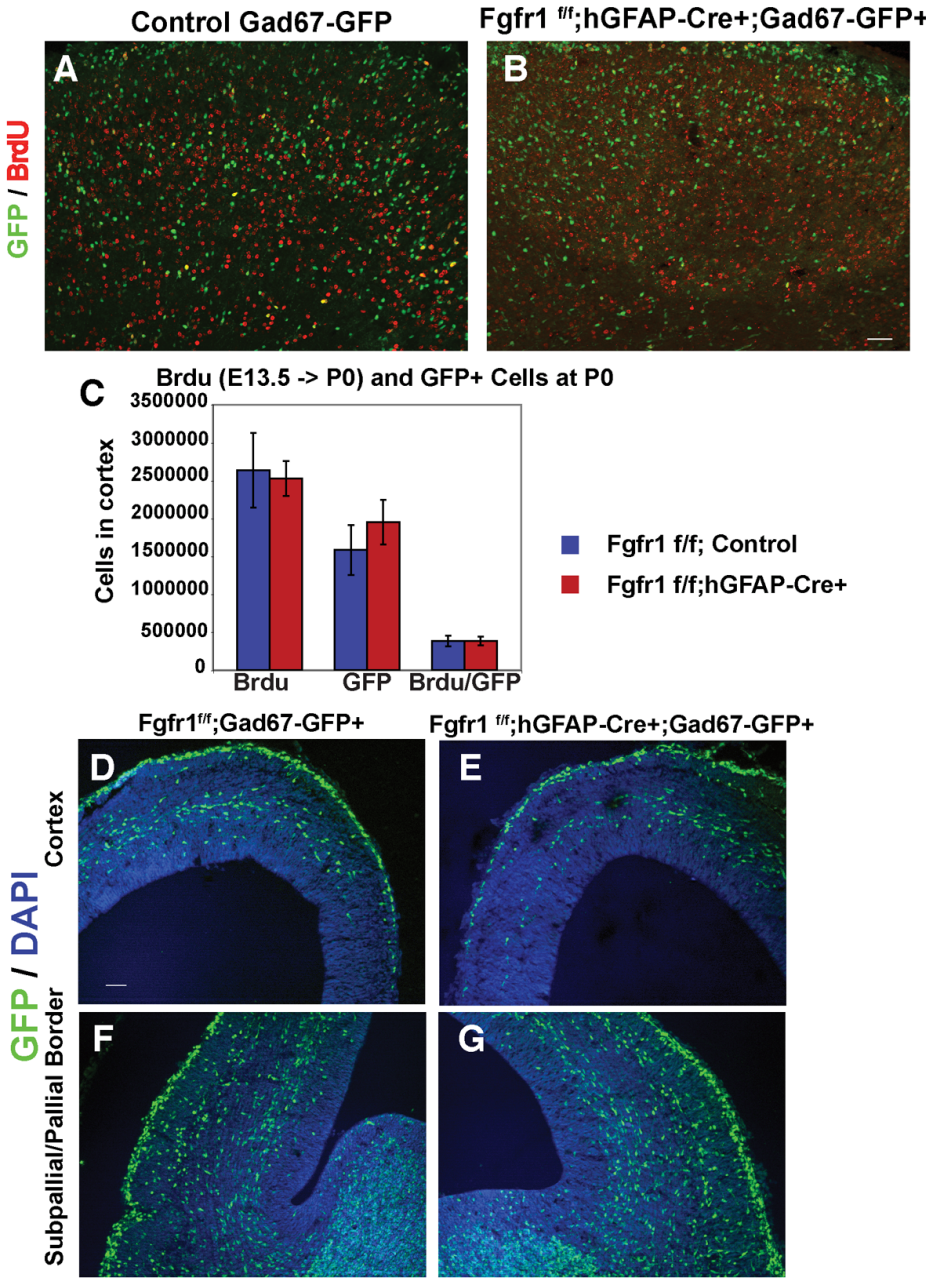
Birthdating and migration studies of cortical Interneurons genetically labeled with GFP reveal no differences in the initial birth and migration of interneurons in Fgfr1 mutant mice. BrdU (100 mg/kg at E13.5) and GFP labeling in the cortex of P0 *Fgfr1^f/f^;Gad67-GFP+* control (**A**) and *Fgfr1^f/f^;hGFAP-Cre;Gad67-GFP+* mutant mice (**B**). No significant differences in total BrdU single labeling, GFP single labeling, or Brdu/GFP double labeling were observed at P0 (n = 4 for controls, n = 3 for Fgfr1 mutants (**C**). Coronal sections visualizing migrating cortical interneurons at E14.5 in *Fgfr1^f/f^;Gad67-GFP+* control (**D**, **F**) and *Fgfr1^f/f^;hGFAP-Cre;Gad67-GFP+* mutant (**E**, **G**) embryos. The interneurons cross the subpallial/pallial border (compare **F** and **G**), appear to reach the equivalent position in the cortex and travel the characteristic route of migration in control and mutant mice (compare **D** and **E**). Scale bar is 50 µm in B (**A** and **B**). Scale bar in D is 50 µm in D (**D**–**G**).

We then asked whether GABAergic progenitor cell survival was altered by the absence of Fgfr1 signaling in neural progenitors by fate mapping progenitors with BrdU. In this experiment, interneurons were labeled genetically with the Gad67-GFP allele. Pregnant dams (E13.5) were injected with 100 mg/kg of BrdU and pups were harvested at P0. We compared the cortex of mutant *Fgfr1^f/f^;hGFAP-Cre+;Gad67-GFP+* to control littermates *Fgfr1^f/f^;Gad67-GFP+*. No differences in total BrdU+ cells, or in BrdU+/GFP+ cells were observed in the cerebral cortex between mutant and control mice (**Figure 4A–C**). The data suggest that the same number of BrdU labeled interneurons, born at E13.5, were present in the cortex of Fgfr1 mutant animals at birth. Furthermore, we found no differences in the number of GFP+ neurons in the cortex of *Fgfr1^f/f^;hGFAP-Cre+;Gad67-GFP+* mice at P0 (**Figure 4C**), suggesting that the decrease in PV+ interneurons is likely to occur postnatally.

**Figure 5:**
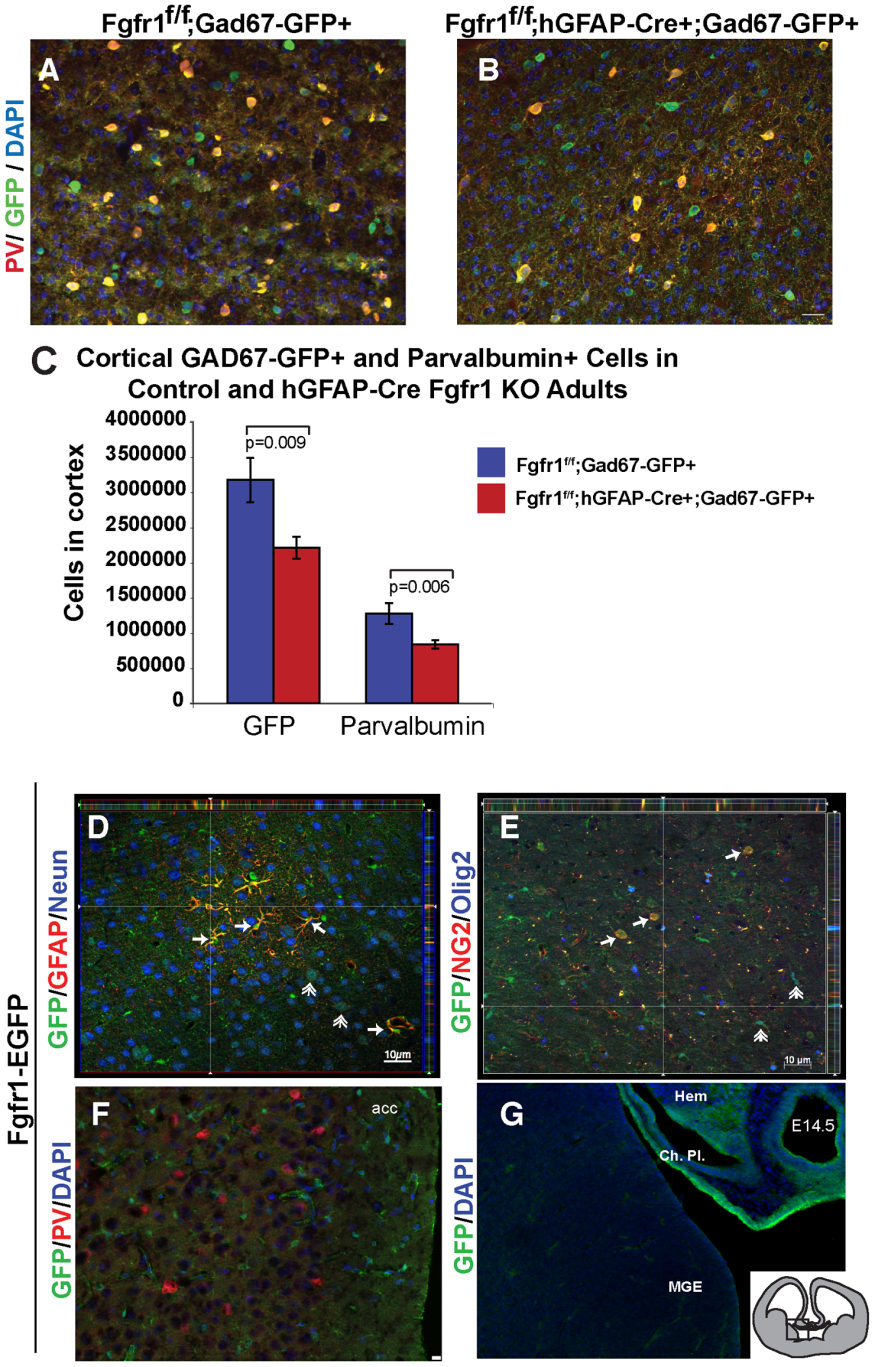
Postnatal decrease in PV+ cortical interneurons in Fgfr1 mutant mice. PV and GFP immunostaining in 8 week old *Fgfr1^f/f^;Gad67-GFP+* control (**A**, n = 5) and *Fgfr1^f/f^;hGFAP-Cre;Gad67-GFP+* mutant mice (**B**, n = 9). Scale bar is 50 µm in **A**,**B**. Stereological analysis show significant decreases in in both GFP and PV immunopositive cells in Fgfr1 mutant mice as compared to littermate controls (**C**). Immunostaining for GFAP, Neun, Ng2, Olig2 and GFP in cortex of adult TgFgfr1-GFP mice (**D, E, F**). Cortical GFAP+ cells colocalized with strong GFP expression (arrows), while some Neun+ cells (double arrows) also colocalized with weak GFP (**D**). NG2 positive cells (arrows), and some Olig2 positive cells (double arrows) also colocalized with weak GFP (**E**). PV immunoreactivty did not colocalize with GFP (**F**, scale bar is 10 µm and 20 µm in **G**). In embryonic brain, GFP was highly expressed in the hippocampal Hem and choroid plexus, but was very low in the MGE (**G**, insert shows coronal section and region imaged). acc = anterior cingulate cortex acc, MGE = medial ganglionic eminence, Hem = hippocampal hem, Ch. Pl = choroid plexus.

GABAergic interneurons migrate from the GE to the cortex via tangential migration. We examined the migration of interneurons in coronal sections at E14.5 in *Fgfr1^f/f^;hGFAP-Cre+* mutant and control mice both in the *Gad67-GFP* background. Migration of interneurons appeared preserved in Fgfr1 mutant mice (**Figure 4D,E**) compared to littermate controls (**Figure 4F,G**), consistent with the finding of no altered number of GFP+ cells in the cortex of Fgfr1 mutant animals at P0. To confirm that the presence of the Gad67-GFP allele does not interfere with the adult phenotype we examined GFP+ and PV+ interneurons in adult *Fgfr1^f/f^;hGFAP-Cre+* and control mice in the *Gad67-GFP* background (**Figure 5A,B**). Using stereology, we found a 30% decrease in GFP+ positive cells, and a 35% decrease in PV positive cells in 8 week old *Fgfr1^f/f^;hGFAP-Cre+;Gad67-GFP+* mice compared to their control *Fgfr1^f/f^;Gad67-GFP+* littermates (**Figure 5C**). These results are comparable to those obtained in mice without the *Gad67-GFP+* allele [9].

Both the hGFAP-Cre and Nestin-Cre trangenes are expected to recombine floxed alleles of *fgfr1* in both cortical excitatory neurons and in cortical astrocytes. In order to determine which cortical cell types express the Fgfr1 gene, we examined the Tg(Fgfr1-EGFP)GP338Gsat line from GENSAT that expresses GFP from the Fgfr1 promoter of a bacterial artificial chromosome transgene insertion. GFP expression under the Fgfr1 promoter was mostly directed to cortical astrocytes with abundant GFAP/GFP double-labeled cells. Fgfr1 driven weak GFP expression was also observed in some neurons (Neun), Oligodendrocytes (Olig2), and NG2 positive cells (**Figure 5D,E**). Since a small percentage of Neun positive cells express GFP, we examined whether these cells are PV positive. PV and GFP immunostaining did not colocalize in the young adult (1 MO) cortex (**Figure 5F**) demonstrating that PV neurons do not likely express Fgfr1. Fgfr1-EGFP embryos at E14.5 showed high expression of GFP in the hippocampal hem and choroid plexus, as expected from in situ hybridization data. However, very little GFP signal was visible in the MGE (**Figure 5G**).

### Analysis of apoptotic cells in early postnatal Fgfr1 mutant animals

**Figure 6:**
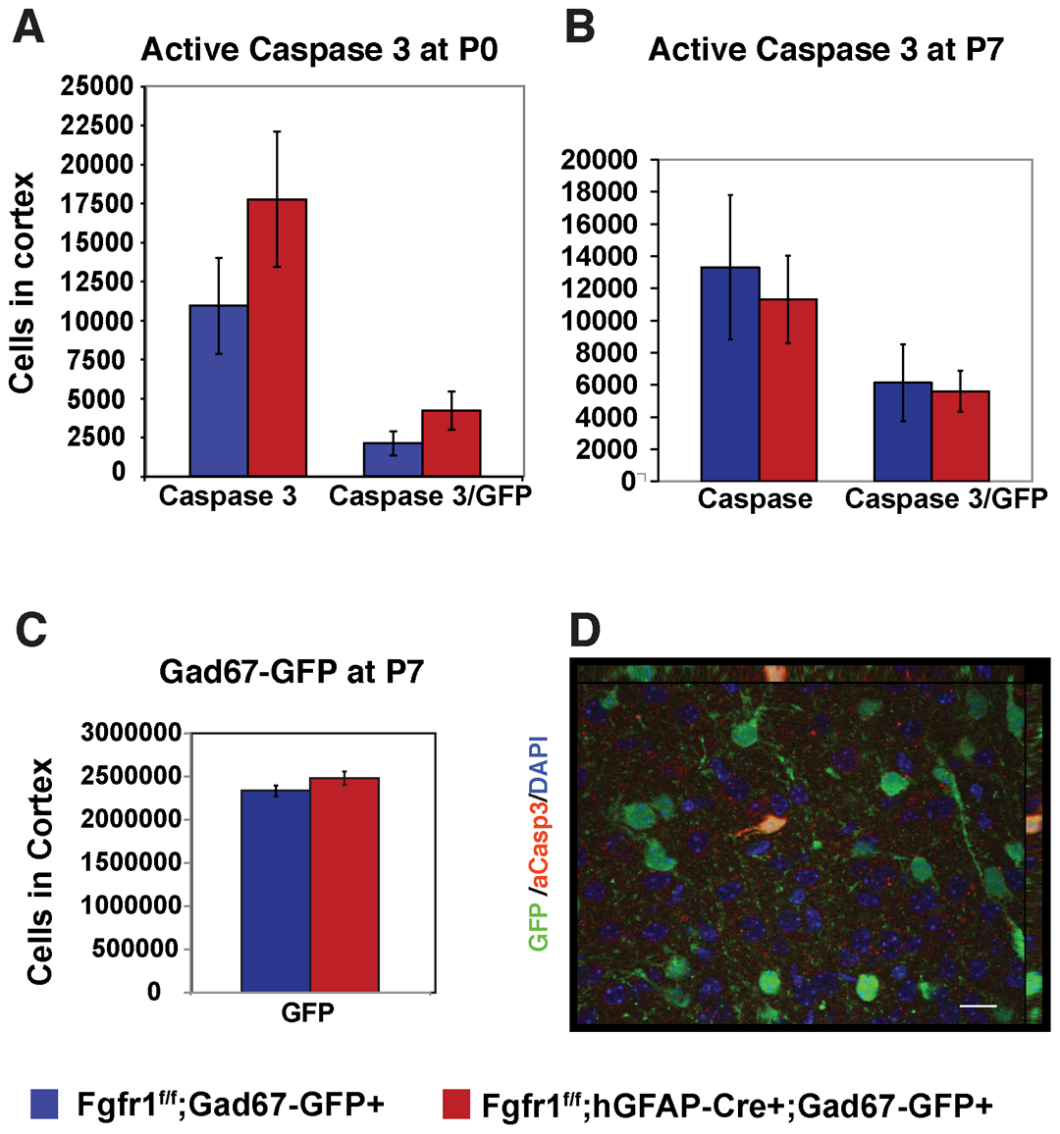
No differences in apoptotic cell death in postnatal interneurons at P0 or P7 of Fgfr1 mutant mice. No differences in total Active Caspase 3 positive cells, or in GFP/Active Caspase 3 double positive cells were observed in *Fgfr1^f/f^;Gad67-GFP+* control and *Fgfr1^f/f^;hGFAP-Cre;Gad67-GFP+* mutant mice at P0 (**A**, n = 4 controls, n = 3 Fgfr1 mutants) or at P7 (**B**, n = 4 controls, n = 5 Fgfr1 mutants). No differences in the number of GFP positive interneurons were observed at P7 (**C**), indicating that the loss of GFP staining seen in adult animals is occurring after P7. Representative staining of activated caspase 3 and GFP (**D**). Scale bar is 5 µm.

Given that Fgfr1 mutant animals have normal numbers of GFP+ interneurons at birth, and that this number is significantly decreased by 8 weeks of age, we examined whether this decrease is due to cell loss by apoptotic cell death. To examine this possibility, we examined the number of Active Caspase 3+ and *Gad67-GFP+* cells at P0 and at P7. A previous study demonstrated that cortical interneuron apoptosis peaks at P7 [22]. At P0 we observed a non-significant trend (p = 0.10) towards increased numbers of active Caspase 3+ cells single and double labeled with GFP in the cortex of *Fgfr1^f/f^;hGFAP-Cre+;Gad67-GFP* as compared to control mice (**Figure 6A**). At P7 we saw no significant differences in active Caspase3+ cells, active Caspase 3 and GFP double labeled cells, or in GFP+ GABA interneurons between mutant and control mice (**Figure 6B–D**). The observation that GFP+ GABA interneurons are unchanged at this age indicates that either the decrease in GFP+ interneurons observed at 8 weeks is occurring after P7, or that the cells are not lost by cell death, but rather fail to mature properly.

### Astrocyte mediated support of interneurons is impaired in Fgfr1 mutant animals

Previous reports, and our findings with the tgFgfr1-EGFP mice, show that Fgf receptors including Fgfr1 are expressed in astrocytes postnatally [23]–[25]. Fgf signaling is known to promote glial cell fates. Therefore, we hypothesize that the loss of Fgfr1 in astrocytes in the postnatal period is resulting in a cortical environment that is less capable of supporting the survival or maturation of cortical interneurons. To test these hypotheses, we examined by immunoblotting several candidate molecules expressed by neurons or glia, which could potentially be involved in altered astroglia-neuron interactions in Fgfr1 mutants, and which may be important for neuronal survival or interneuron maturation. We found no differences in the amount of GLAST, HGF, or BDNF in the cortex of *Fgfr1^f/f^;Nestin-Cre+* mice compared to littermate controls (**Figure S1C**,**D**).

**Figure 7:**
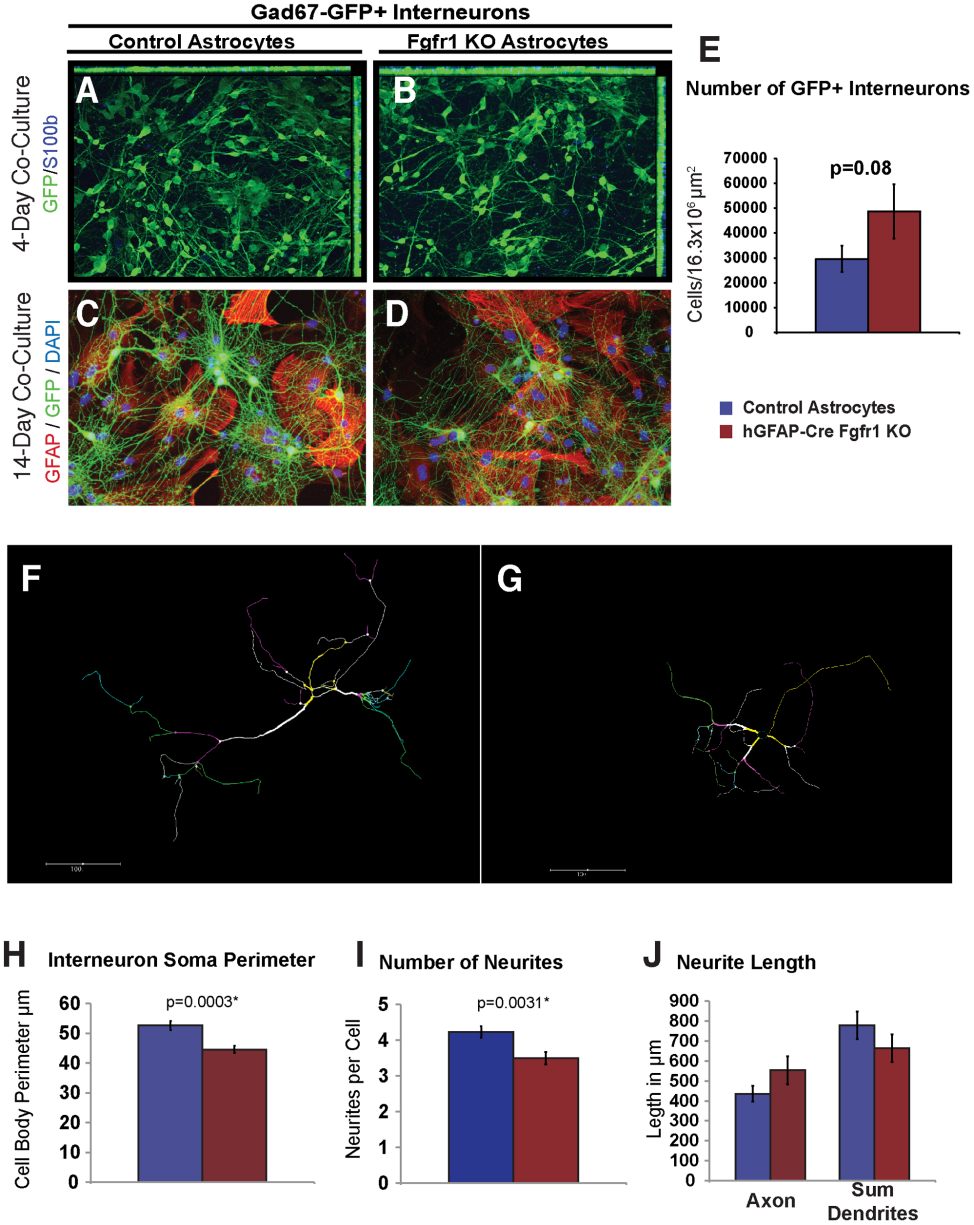
Astrocyte-Interneuron co-culture experiments reveal altered cytoarchitecture for interneurons grown on astrocytes lacking Fgfr1. *Gad67-GFP+* positive interneurons were isolated from the MGE of E13.5 embryos wild type for Fgfr1, and cultured on astrocytes from *Fgfr1^f/f^* control (**A**, **C**) or *Fgfr1^f/f^;hGFAP-Cre* mice (**B**, **D**). Co-cultures maintained for 4 days had abundant numbers of GFP positive cells that appeared to be immature, with few processes (**A**,**B**, green = GFP, blue = s100beta). After two weeks in culture, interneurons developed many branched neurites, and appeared to prefer attachment to GFAP (red) positive areas of the culture dish. After two weeks in culture, no differences in the number of surviving interneurons were observed (**E**, averaged from 26 culture wells of 5 controls, and 12 culture wells of three Fgfr1 mutant mice). Representative Neurolucida drawings showing decreased complexity of interneurons grown on Fgfr1 mutant astrocytes as compared to those grown on control astrocytes (**F** and **G**). Cell perimeter (**H**) and the number of neurites per cell (**I**) were decreased based on neurolucida drawings of 60 neurons from 5 control samples, and 38 neurons from three Fgfr1 mutant samples. Axon length or the sum of dendrite lengths were not significantly different between the two groups (**J**).

We then developed an *in vitro* co-culture system in order to test the ability of astrocytes lacking Fgfr1 to support the survival and maturation of cortical interneurons derived from MGE precursors. Cortical astrocyte monolayers were obtained from the cortex of early postnatal (P2–P4) *Fgfr1^f/f^;hGFAP-Cre+* and *Fgfr1^f/f^* control littermates. Interneuron precursors were isolated from the MGE of *Gad67-GFP+* mice wild type at the *fgfr1* locus at E13.5 and plated upon the astrocyte monolayers. Co-cultures were maintained for 15 days, and stained for GFAP and GFP. After 4 days in culture, interneurons appeared to have simple morphology (**Figure 7A,B**), while after 15 days in culture, larger cell bodies with numerous neurite branches with complex morphology were observed (**Figure 7C,D** **)**. The number of astrocytes and interneurons was estimated by random sampling of sites on the culture slide well by using the fractionator mode of StereoInvestigator. There was a trend towards an increase in the number of surviving GFP+ interneurons in neurons co-cultured with astrocytes derived from Fgfr1 mutants and control (**Figure 7C–E**). However, the morphology of interneurons growing on mutant astrocytes was abnormal, as reveled by reconstruction of randomly selected GFP+ interneurons, and analysis of cell area and branch structure performed with the Neurolucida module from Microbrightfield (representative drawings are shown in **Figure 7F and G**). ANOVA analysis of Neurolucida measures revealed that cell perimeter size, F(1,97) = 13.85, p = 0.0003, and the number of neurites per cell, F(1,97) = 2.51, p = 0.0031, were significantly decreased in interneurons co-cultured with astrocytes lacking Fgfr1, compared to astrocytes from control mice (**Figure 7H,I**). The length of axons and the sum dendrite length were not altered by co-culture with Fgfr1 mutant astrocytes (**Figure 7J**). Post hoc analysis revealed that cell perimeter size and the number of neurites per cell were significantly correlated to each other, R^2^(97) = .11, p<0.001. To determine whether there was a group effect upon cell body perimeter independent of the number of neurites, an analysis of covariance was performed. Both the number of neurites, t-ratio 2.64, p = 0.01, and genotype group, t-ratio = 2.88, p = 0.005, were significant, indicating independent effects of genotype on both measures. Parallel astrocyte-interneuron co-cultures were maintained for 21 days with induction of PV expression achieved by addition of a subdepolarizing potassium chloride concentration to the culture media. PV expression was not affected by co-culture with astrocytes from Fgfr1 mutant animals *in vitro* (**Figure S3**). These results indicate that Fgfr signaling in astrocytes may play a role in cell trophism (as shown by cell body size and number of neurites) and maturation of their normal cytoarchitecture.

**Figure 8:**
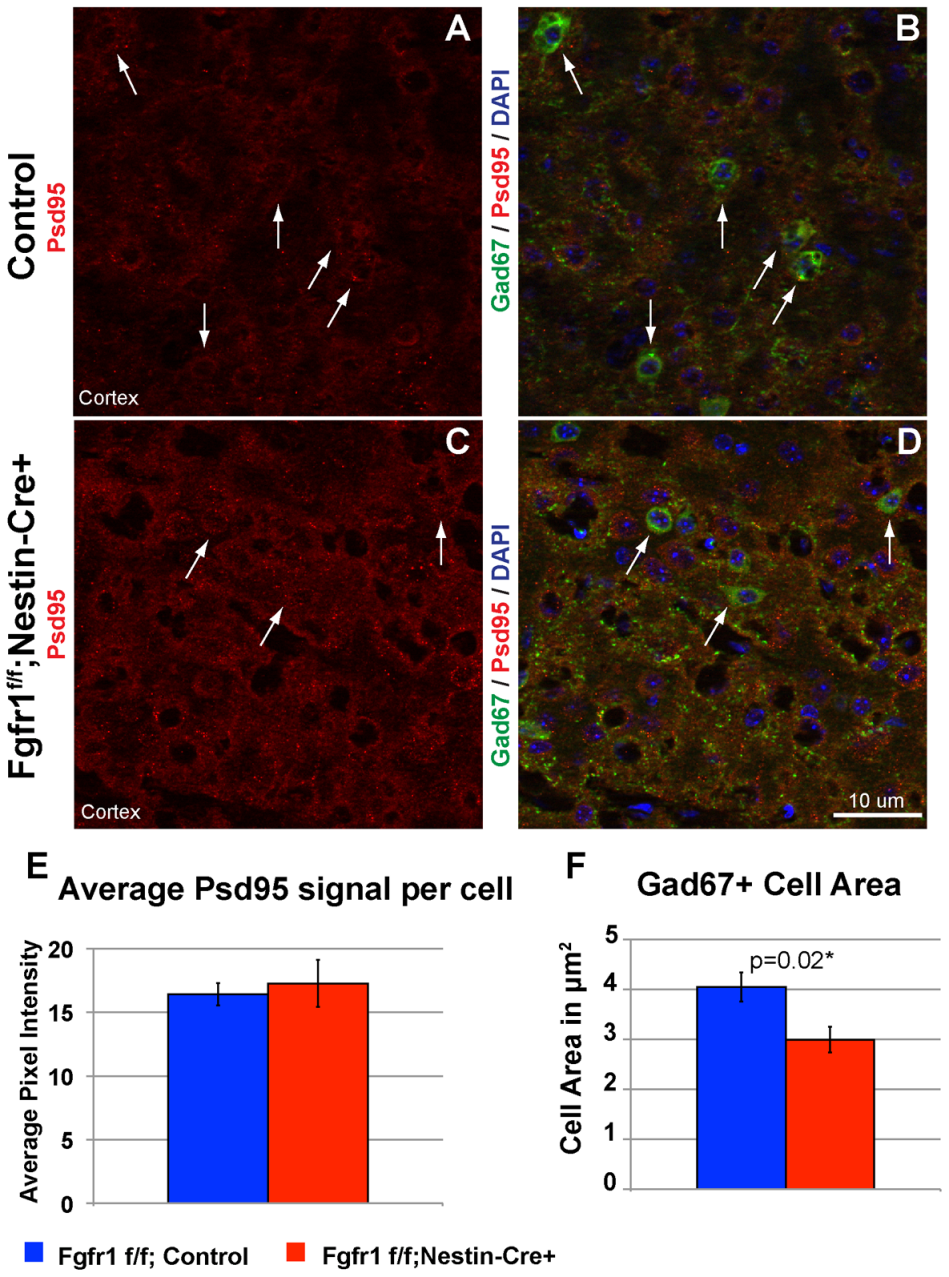
Decreased interneuron soma size in mice lacking Fgfr1. Immunostaining for Post Synaptic Density 95 (PSD95, red in **A**–**D**) and Gad67 enzyme (green in **B** and **D**) in *Fgfr1^f/f^* control (**A**,**B**) and *Fgfr1^f/f^;Nestin-Cre* (**C**–**D**) mice. Arrows, GAD67 positive cells. Interneurons were identified via GAD67 staining, circled and circled area was analyzed for intensity of PSD95 staining using Image J. No differences in the total PSD95 signal were observed (**E**). The cell perimeter of cortical interneurons stained by Gad67 was significantly decreased in *Fgfr1^f/f^;Nestin-Cre* mice as compared to controls (**F**). Control n = 6, Fgfr1 mutant n = 6.

Previous studies indicate that Fgfr signaling may contribute to synapse development [26], [27]. We examined PSD95 staining upon the cell bodies of Gad67 immunopositive cells in the cortex of one month-old Fgfr1 mutant animals (*Fgfr1^f/f^;Nestin-Cre+*). We found no differences between control and Fgfr1 mutant animals in the average intensity of staining for PSD95 upon Gad67 immunopositive cell bodies (**Figure 8A–E**). We also examined the cell body perimeter of Gad67 immunopositive cells, and found a statistically significant difference in cell soma size (**Figure 8F**). The soma of GABAergic cells staining for Gad67 was smaller in Fgfr1 mutant cortex, which is similar to our finding in GABAergic interneurons isolated from the MGE and grown upon Fgfr1 mutant astrocytes (**Figure 7**).

## Discussion

Here, we show that inactivation of Fgfr1 in the developing telencephalon results in a deficiency of PV positive interneuron maturation and decreased PV immunoreactivity in the postnatal period. Adult mice with Fgfr1 mutations driven by the Nestin-Cre and hGFAP-Cre transgenes have a deficit in PV+ interneurons in the cortex. Proliferation of interneuron precursors in the GE was not affected in mice with either Nestin-Cre-, or hGFAP-Cre-driven mutation of Fgfr1. Fgfr1 mutant mice are born with the same number of cortical interneurons at P0, and have the same number of cortical interneurons at P7. No differences in apoptotic cells were observed at these two perinatal ages. Therefore, a reduction in PV and Gad67-GFP immunostaining must be occurring at an intervening time period, perhaps in the juvenile or adolescent period.

Fgfr1 is expressed in astroglia postnatally, as shown by GFP expression driven by the Fgfr1 promoter in the Tg(Fgfr1-EGFP)GP338Gsat mice. In further support of the astrocyte expression of Fgfr1, both Fgfr1 and Fgfr3 are listed among 311 genes that have enriched expression in astrocytes as compiled by Network Glia organization (http://www.networkglia.eu/en/astrocyte). This list was assembled from three independently reported peer reviewed publications [28]–[30].

Given this predominant astrocyte expression, it was surprising that Nestin-Cre driven Fgfr1 knockout was more effective than that driven by hGFAP-Cre at reducing PV neuron number. However, the hGFAP-Cre mediated recombination likely does not target all cortical astrocytes, as the human GFAP promoter fragment used in the hGFAP-Cre transgenic mice is active in only a portion of cortical astrocytes [31]. The heterogeneity of astroglial cells with regards to GFAP expression is well documented in the literature. Presumably, Nestin-Cre mediated recombination not only starts earlier but it is also more widespread than that of hGFAP-Cre. Hence, it is reasonable to assume that inactivation of Fgfr1 via Cre mediated recombination is more complete in Nestin-Cre animals. It is also clear that Nestin-Cre animals would more extensively target the progenitors of PV+ interneurons in the MGE. However, a very low level of Fgfr1 expression was detected in the embryonic MGE and no expression of Fgfr1 was detected in postnatal PV+ cells using the Tg(Fgfr1-EGFP)GP338Gsat transgenic line.

PV+ interneurons are born at approximately E12.5 to E15.5 in the medial ganglionic eminence of the ventral telencephalon, and migrate tangentially to the dorsal cortex where they integrate into the cortical circuitry [32]–[38]. The migration and integration of cortical interneurons is ongoing at the time of birth, as is the maturation of interneuron identity [32]. PV immunoreactivity is upregulated in the first few weeks of postnatal life [39]–[41] in concomitance with their extended period of postnatal maturation. Expression of PV protein by immunohistochemistry is not detectable until approximately 12–14 days after birth in the mouse. This postnatal maturation period coincides temporally with the periods of plasticity in the sensory cortex, and has been implicated in the establishment of critical periods for visual system development. Interestingly, experiments with NMDA receptor antagonist MK801 have also revealed this to be a time of vulnerability for PV+ interneurons [42], [43]. The KV3.1b potassium channel that confers fast spiking properties to PV+ basket cells and chandelier neurons is also upregulated in the maturing cortex during the juvenile to young adult stages [19], [20].

Several lines of evidence suggest that PV neuron maturation, but not survival, is impaired in Fgfr1 mutants. First, other markers of PV interneurons were not significantly decreased at the protein or mRNA level, in the presence of a consistent decrease in PV immunoreactivity. For example, no differences in KV3.1b mRNA and protein were found in Fgfr1 mutant animals, although this could also be attributable to a wider expression of this channel by other neurons besides PV+ interneurons.

In addition to PV, Fgfr1 mutants have decreases in both Gad67 immunoreactive cells and in GFP expression driven by the Gad1-GFP allele *in vivo*. This is consistent with a maturation defect in these animals, as Gad67 expression is prominently upregulated in the early postnatal period, concomitantly with PV [44]. The fact that calretinin positive and somatostatin positive neurons were not reduced, and that Lhx6 and PV mRNA were not decreased in Fgfr1 mutant animals, all point to a defect in the maturation of cortical interneurons as opposed to their loss by necrosis or apoptosis. Using an *in vitro* co-culture system, we tested the hypothesis that astrocytes from Fgfr1 mutants are less capable of supporting the survival and maturation of interneurons than astrocytes from control animals. While survival of interneurons and length of processes was unaffected in interneurons co-cultured with Fgfr1 mutant astrocytes, the cell soma size and number of neurites were significantly reduced. A similar decrease in cell body size was observed in Gad67+ interneurons in the cortex of Fgfr1 mutant mice. These data confirm that the trophic effect of astrocytes is impaired in Fgfr1 mutant astrocytes, affecting the maturation of the interneurons.

Previously, Fgfr1 and Fgfr2 double mutants generated with the FoxG1-Cre driver were shown have disrupted MGE formation [21]. However, we did not detect such changes in Fgfr1 mutants (or in Fgfr1/Fgfr2 double mutants) generated with the hGFAP-Cre or Nestin-Cre drivers, presumably because of later onset of Fgfr recombination. This agrees with our findings that the loss of MGE-derived interneurons in our Fgfr1 mutants does not occur in the embryonic period, but postnatally. The latest time point examined where no changes in interneuron number was observed was P7, and the earliest time point examined, where a decrease in PV+ interneurons was observed was P49. Therefore, the reduction in PV protein levels is occurring within this time frame- the late juvenile and young adult time period, when cortical interneurons attain their full maturation, and when they are vulnerable to damage by the NMDA antagonist MK801.

PV neurons play a crucial role in cortical information processing. The axonal outputs of PV+ basket cells target the soma of excitatory neurons, while PV+ chandelier interneurons target the initial axonal segment of excitatory neurons [12], [45]. This property confers the ability to regulate the output of excitatory neuron firing. For example, paired recordings of hippocampal PV neurons and excitatory neurons show that PV neurons firing in a theta rhythm entrain excitatory neurons to the same rhythm [46], [47]. Furthermore, fast-spiking PV+ chandelier cells in the cortex are thought to modulate and entrain the firing of cortical excitatory projection neurons within cortical columns, increasing the ability of these active columns to send inputs to the basal ganglia and thalamus [12], [16], [48].

A similar decrease in the maturation of PV interneurons in the cortex is caused by chronic perinatal hypoxia, which models prematurity [49]. The loss or dysfunction of GABAergic interneurons has been implicated in various psychiatric disorders [12], [50]–[56]. Early studies of mRNA expression in post mortem tissue identified a decrease in Gad1 mRNA (coding for the Gad67 protein) in the prefrontal cortex of individuals with schizophrenia or bipolar disorder [57], [58]. Follow up studies have confirmed changes in the expression in Gad1 mRNA, and other GABAergic molecules such as PV, Sst, and NPY, in individuals with psychosis [44], [57], [59]–[61]. The density of nonpyramidal neurons, in particular, PV+ and calbindin+ interneurons, in the anterior cingulate and PFC of individuals with schizophrenia or bipolar disorder is also reduced, suggesting a loss or impairment of GABAergic neurons in these disorders [62], [63]. Reduced GABA concentrations have been reported in plasma, cerebrospinal fluid, and cortex of depressed subjects, while proton magnetic resonance spectroscopy have also found a decrease in GABA in the occipital cortex of depressed individuals [64]–[66]. Reductions in PV neurons have been observed in various other animal models for schizophrenia or anxiety [9], [67]–[71]. Animal models such as the Fgfr1 mutation, that result in postnatal deficiencies in cortical interneuron number or maturation, may be useful in studying mechanisms of interneuron dysfunction in these disorders, and for formulating new strategies whereby interneuron maturation and survival can be augmented.

Fgfr1 is expressed in glial cells at the time that the decrease in interneuron number is occurring. Although an indirect role of excitatory neurons in the development of inhibitory neurons cannot be ruled out, by PSD95 staining within inhibitory neurons, this measure of direct synaptic contact between these neuronal populations was found to be normal. Therefore, we hypothesize that Fgfr1 is needed within postnatal astrocytes for promoting intercellular signaling that supports the maturation and/or survival of cortical interneurons. FGFR signaling has been shown to upregulate the expression of GFAP in astrocytes [24], [72]; however, no major effect on GFAP expression was noted in Fgfr1 mutant animals. Astroglia are essential for maintaining the health and function of neurons by a multitude of mechanisms including glutamate and GABA homeostasis via the glutamate-glutamine-GABA cycle [73]. Astrocytes maintain the blood brain barrier, which may also regulate the entry of glucose into the neurons [72], [74]. Astrocytes have an additional role in neuronal bioenergetics, by providing neurons with a crucial metabolite, lactic acid (reviewed by [74], [75]). This pathway may be particularly important for neurons with high energetic requirements, such as fast firing PV+ interneurons. Furthermore, astrocytes maintain ionic and osmotic homeostasis in the CNS by buffering excess potassium ions and water through inward rectifying potassium channels and aquaporin channels, respectively. Lastly, astrocytes can influence nearby neurons by secretion of cytokines, such as FGF, or trophic factors such as glial derived neurotrophic factor which promote protein synthesis in target cells (reviewed by [74], [76]). When cortical interneurons isolated from the MGE were grown upon astrocytes from Fgfr1 mutant animals, we observed a smaller cell soma size and fewer neurites, possibly reflecting differences in the trophic role of astrocytes upon neurons. To better understand these effects, future experiments should elucidate the effect of FGF signaling on the astrocyte transcriptome and physiology.

## Supporting Information

Figure S1 **Representative western blot of** ***Fgfr1^f/f^*** **control and** ***Fgfr1^f/f^;Nestin-Cre*** **animals (7 week old) for PV and beta actin loading control (A).** Comparison of band intensity values for PV (normalized for beta-actin) in control (n = 6) and Fgfr1 mutant (n = 6) animals (**B**). Representative western blots for KV3.1b, BDNF and beta actin (**C**) as well as HGF, GLAST and beta actin (**D**) reveal no differences in the levels of these proteins involved in interneuron maturation (KV3.1b, BDNF, HGF) or glial function (HGF, GLAST).
(TIF)

Figure S2 **Expression of KV3.1b in Fgfr1^f/f^ control and Fgfr1^f/f^; Nestin-Cre+ mutants.** Immunostaining of cingulate cortex revealed that the KV3.1b antibody (**A, E**) co-localizes with PV (**B, F**) in the cortex (merged images **C,G**). However, contrary to previous reports, we see various KV3.1b positive cells, that do no express PV. These cells are present in both control and Fgfr1^f/f^; Nestin-Cre+ mutants. Immunofluorescence for Sst in control (**D**) and Fgfr1^f/f^; Nestin-Cre+ mutants (**H**) was also performed, and no significant difference in cortical cell number was observed (**I**). QRT-PCR for KV3.1b was performed in young adult mice (one-month, **J**) and in adult mice (4 month, **K**), and for Lhx6 (**L**), PV (**M**), and Fgfr1 (**N**) in adult mice (4 months).
(TIF)

Figure S3 **Maintenance of cells for 21 days in culture in the presence of 25 mM potassium chloride resulted in some cells gaining PV staining (A,B).** The number of PV+ cells did not differ between control and *Fgfr1^f/f^;hGFAP-Cre* mice (**C**, count on 12 culture wells from 3 control samples, and 19 culture wells, from 6 Fgfr1 mutant samples).
(TIF)
