# Supplementary figures and images for: Fgfr1 Inactivation in the Mouse Telencephalon Results in Impaired Maturation of Interneurons Expressing Parvalbumin
Source: PLoS One. 2014 Aug 12;9(8):e103696. doi: 10.1371/journal.pone.0103696 (PMC4130531; doi:10.1371/journal.pone.0103696)

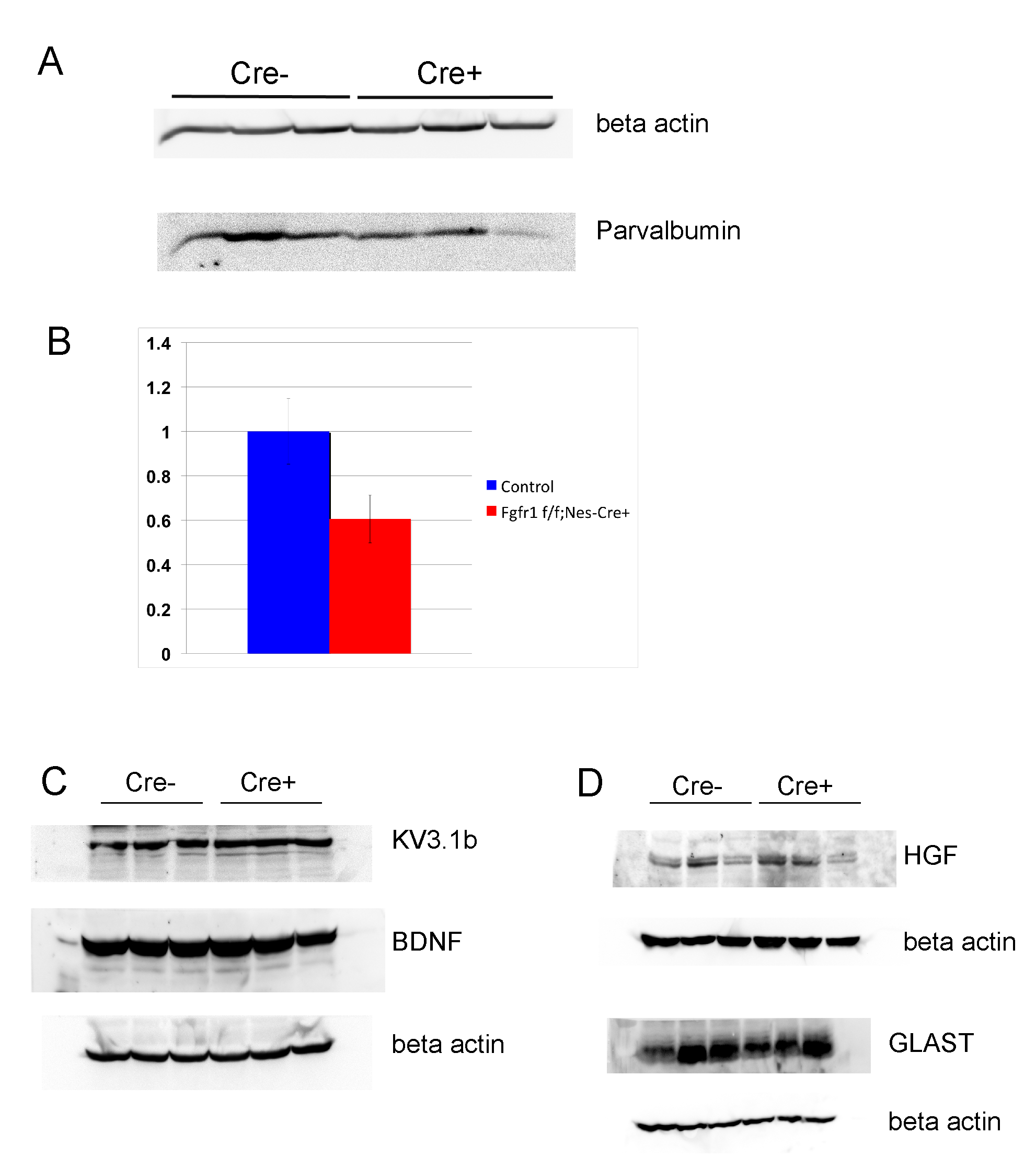

Supplement: Figure S1 — Representative western blot of Fgfr1f/f control and Fgfr1f/f;Nestin-Cre animals (7 week old) for PV and beta actin loading control (A). Comparison of band intensity values for PV (normalized for beta-actin) in control (n = 6) and Fgfr1 mutant (n = 6) animals (B). Representative western blots for KV3.1b, BDNF and beta actin (C) as well as HGF, GLAST and beta actin (D) reveal no differences in the levels of these proteins involved in interneuron maturation (KV3.1b, BDNF, HGF) or glial function (HGF, GLAST). (TIF) [file pone.0103696.s001.tif]

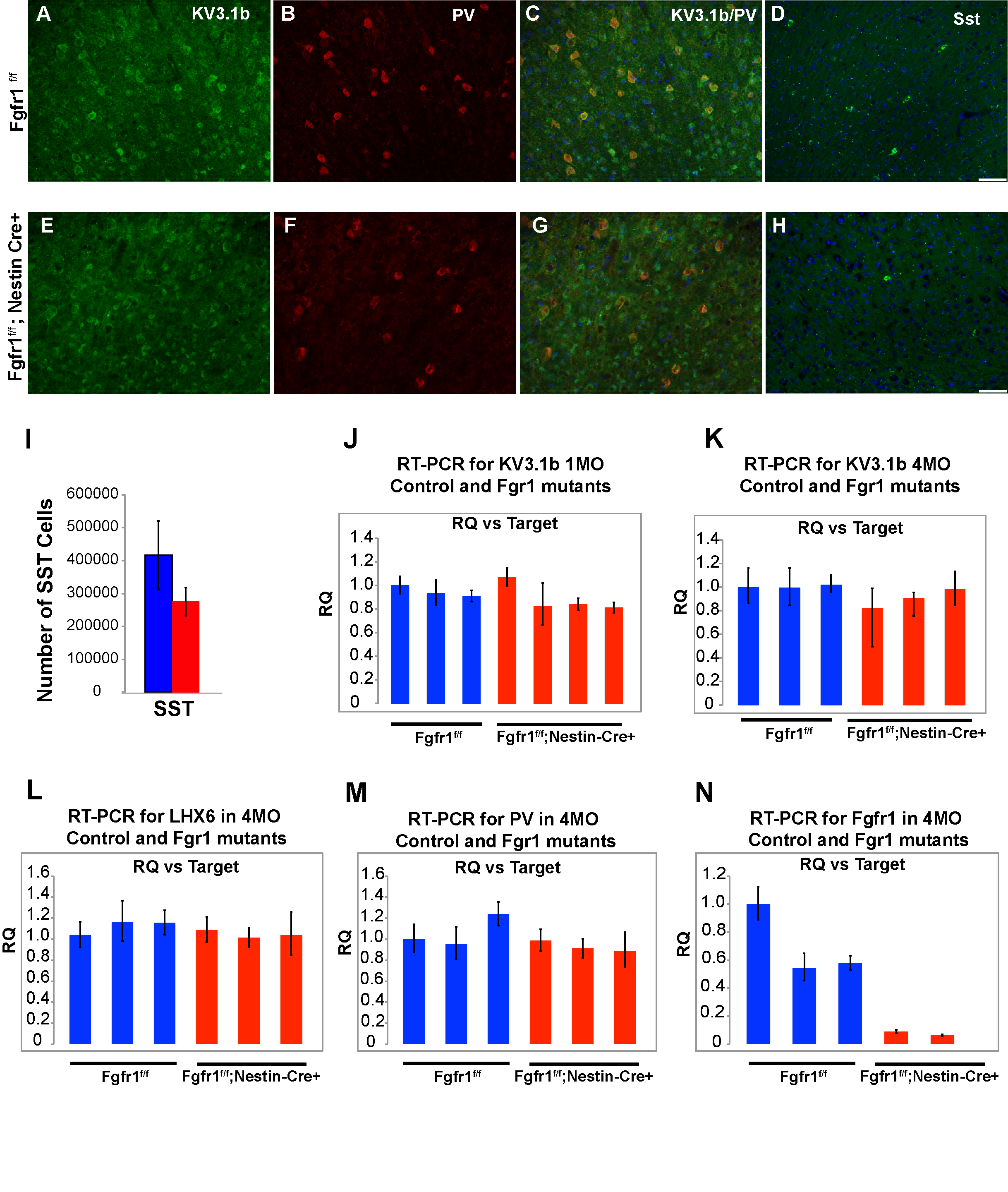

Supplement: Figure S2 — Expression of KV3.1b in Fgfr1f/f control and Fgfr1f/f; Nestin-Cre+ mutants. Immunostaining of cingulate cortex revealed that the KV3.1b antibody (A, E) co-localizes with PV (B, F) in the cortex (merged images C,G). However, contrary to previous reports, we see various KV3.1b positive cells, that do no express PV. These cells are present in both control and Fgfr1f/f; Nestin-Cre+ mutants. Immunofluorescence for Sst in control (D) and Fgfr1f/f; Nestin-Cre+ mutants (H) was also performed, and no significant difference in cortical cell number was observed (I). QRT-PCR for KV3.1b was performed in young adult mice (one-month, J) and in adult mice (4 month, K), and for Lhx6 (L), PV (M), and Fgfr1 (N) in adult mice (4 months). (TIF) [file pone.0103696.s002.tif]

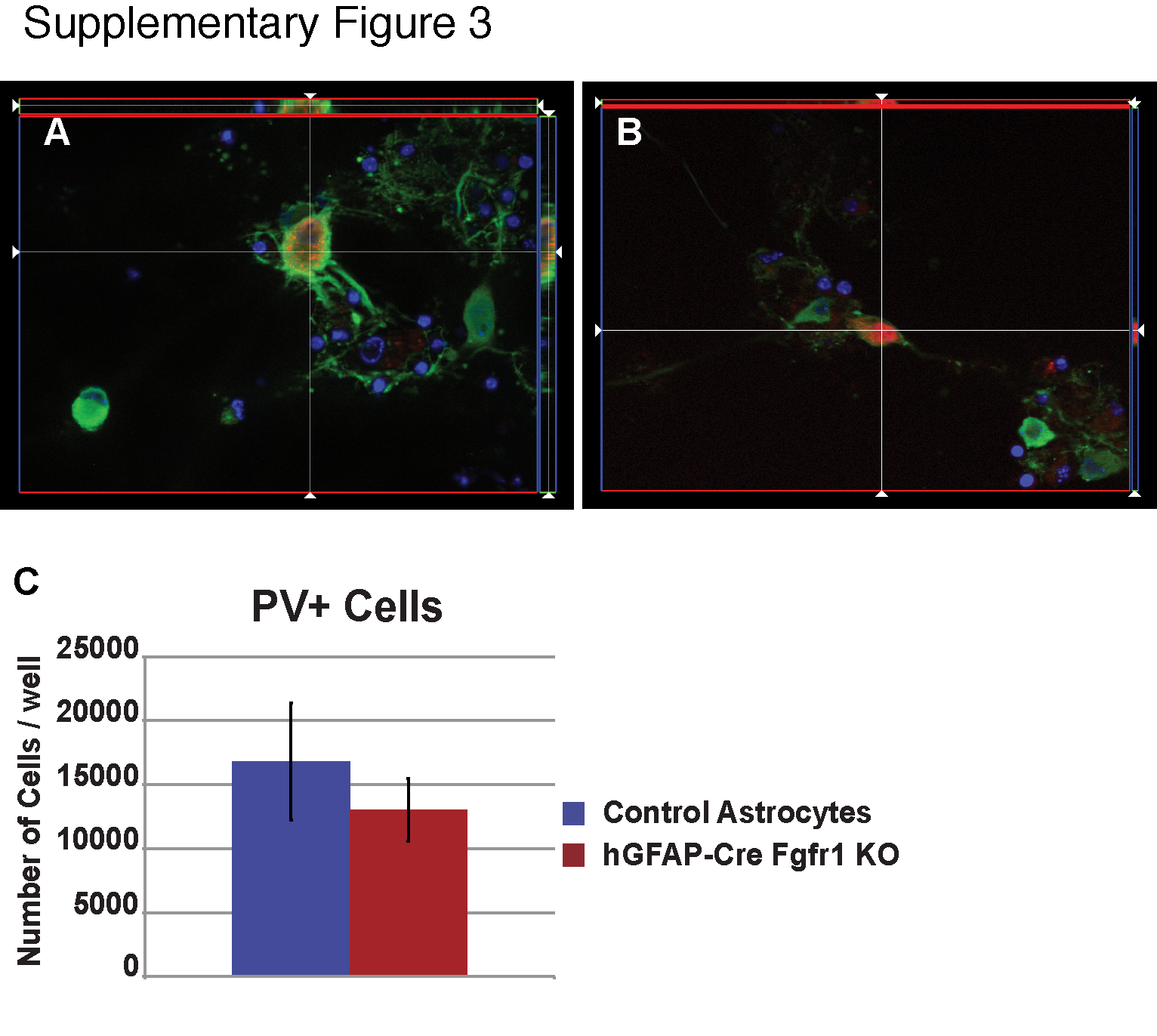

Supplement: Figure S3 — Maintenance of cells for 21 days in culture in the presence of 25 mM potassium chloride resulted in some cells gaining PV staining (A,B). The number of PV+ cells did not differ between control and Fgfr1f/f;hGFAP-Cre mice (C, count on 12 culture wells from 3 control samples, and 19 culture wells, from 6 Fgfr1 mutant samples). (TIF) [file pone.0103696.s003.tif]
